# Infant formula containing galacto-and fructo-oligosaccharides and *Bifidobacterium breve* M-16V supports adequate growth and tolerance in healthy infants in a randomised, controlled, double-blind, prospective, multicentre study

**DOI:** 10.1017/jns.2016.35

**Published:** 2016-10-28

**Authors:** M. Abrahamse-Berkeveld, M. Alles, E. Franke-Beckmann, K. Helm, R. Knecht, R. Köllges, B. Sandner, J. Knol, K. Ben Amor, A. Bufe

**Affiliations:** 1 Nutricia Research, Utrecht, The Netherlands; 2 NETSTAP e.V., Bochum, Germany; 3 Laboratory of Microbiology, Wageningen University, Wageningen, The Netherlands; 4 Experimental Pneumology, Ruhr-University Bochum, Bochum, Germany

**Keywords:** Infant growth, *Bifidobacterium breve* M-16V, Hydrolysed formula, Synbiotics, Randomised controlled trials, ITT, intention-to-treat, PP, per-protocol, RMMM, repeated-measures mixed model, scGOS/lcFOS, short-chain galacto-oligosaccharides/long-chain fructo-oligosaccharides, SCORAD, SCORing Atopic Dermatitis

## Abstract

The objective of the present study was to evaluate the growth and tolerance in healthy, term infants consuming a synbiotic formula with daily weight gain as the primary outcome. In a randomised, controlled, double-blind, multicentre, intervention study infants were assigned to an extensively hydrolysed formula containing a specific combination of *Bifidobacterium breve* M-16V and a prebiotic mixture (short-chain galacto-oligosaccharides and long-chain fructo-oligosaccharides in a 9:1 ratio; scGOS/lcFOS; synbiotic group), or the same formula without this synbiotic concept for 13 weeks (control group). Anthropometry, formula intake, tolerance, stool characteristics, blood parameters, faecal microbiota and metabolic faecal profile were assessed. Medically confirmed adverse events were recorded throughout the study. Equivalence in daily weight gain was demonstrated for the intention-to-treat (ITT) population (*n* 211). In the per-protocol (PP) population (*n* 102), the 90 % CI of the difference in daily weight gain slightly crossed the lower equivalence margin. During the intervention period, the mean weight-for-age and length-for-age values were close to the median of the WHO growth standards in both groups, indicating adequate growth. The number of adverse events was not different between both groups. No relevant differences were observed in blood parameters indicative for liver and renal function. At 13 weeks, an increased percentage of faecal bifidobacteria (60 *v*. 48 %) and a reduced percentage of *Clostridium lituseburense/C. histolyticum* (0·2 *v*. 2·6 %) were observed in the synbiotic group (*n* 19) compared with the control group (*n* 27). In conclusion, this study demonstrates that an extensively hydrolysed formula with *B. breve* M-16V and the prebiotic mixture scGOS/lcFOS (9:1) supports an adequate infant growth.

Exclusive human milk is the preferred feed for all term newborn infants and provides a complete supply of nutrients to support growth and development in early life. In addition, human milk contains bioactive components that beneficially affect intestinal health, gut microbial colonisation and immune maturation^(^^1^^–^^3^^)^. Because human-milk feeding may not always be possible, human-milk substitutes should provide nutritional and functional properties as close as possible to those of human milk. Currently, infants with a family history of allergic diseases that are not (exclusively) breastfed are recommended to receive a partially or extensively hydrolysed formula with confirmed reduced allergenicity^(^^4^^,^^5^^)^. Supplementation of formulae with ingredients influencing the developing microbiome and potentially the maturation of the neonatal immune system have gained an increasing interest in the prevention of allergic diseases^(^^6^^,^^7^^)^.

Prebiotics, non-digestible food ingredients that selectively stimulate the growth and/or activity of intestinal bacteria that affect health positively^(^^8^^)^, are postulated to have a potential preventive effect on the development of allergy. However, according to the Cochrane review of Osborn & Sinn^(^^9^^)^, the overall level of evidence to substantiate a preventive effect of (any) prebiotics on allergy in healthy and/or high-risk populations is currently insufficient, possibly due to considerable heterogeneity, e.g. type of prebiotic intervention, between studies. A more recent review of Vandenplas *et al*.^(^^10^^)^ concludes that prebiotics are safe and since most studies suggest a trend of beneficial effects, their presence brings formula with respect to functionality closer to their ‘gold standard’, breast milk. In individual studies, a specific mixture of short-chain galacto-oligosaccharides and long-chain fructo-oligosaccharides (scGOS/lcFOS; 9:1 ratio) supplemented to infant formula was found to modulate the microbiota of infants towards a composition with more bifidobacteria and less potential pathogenic bacteria such as clostridia-related species^(^^11^^–^^13^^)^. Moreover, it reduced the number of infectious episodes in healthy and at-risk infants^(^^14^^,^^15^^)^ and due to its modulatory effect on the immune system, reduced the risk of atopic dermatitis and some allergic manifestations during infancy and childhood in infants with a high risk of developing allergy^(^^11^^,^^14^^,^^16^^)^.

A 3-month intervention with a synbiotic formula combining the above-mentioned prebiotic scGOS/lcFOS (9:1) mixture with *Bifidobacterium breve* M-16V, which has potential anti-allergic properties^(^^17^^)^, showed a reduction of the SCORing Atopic Dermatitis (SCORAD) score in infants with IgE-mediated atopic eczema as well as a positive modulation of the gut microbiota^(^^18^^)^. In addition, the prevalence of asthma-like symptoms and asthma medication at 1 year of follow-up was lower in the infants who received the synbiotic formula compared with their placebo counterparts^(^^19^^)^. We anticipated that the presence of this synbiotic concept could increase the established efficacy of a well-tolerated extensively hydrolysed whey-based infant formula for the management of allergic disease^(^^20^^,^^21^^)^. However, first, the nutritional safety and adequacy of the addition of such a new functional ingredient should be demonstrated following stringent evaluation^(^^22^^–^^24^^)^.

This equivalence study was aimed at evaluating the growth and tolerance in healthy infants consuming an extensively hydrolysed formula with the prebiotic scGOS/lcFOS (9:1) mixture with *B. breve* M-16V following 3 months of intervention compared with the same formula without this synbiotic concept with daily weight gain as the primary outcome. As secondary outcomes, the study evaluated other growth parameters, tolerance, safety, faecal microbiota, metabolic faecal profile as well as the presence of atopic symptoms.

## Materials and methods

### Ethics statement

Written informed consent was obtained from all parents before randomisation. All participating centres obtained approval of their independent local ethical review board. The study was conducted in compliance with the principles of the Declaration of Helsinki and Good Clinical Practice (GCP) regulations and with those of the local German laws and regulations.

### Study design

This study was performed as a double-blind, placebo-controlled, randomised prospective nutritional intervention study. The study was designed as an equivalence study to assess growth and tolerance in infants using a formula supplemented with a specific combination of pre- and probiotics following 3 months of intervention in healthy infants. The study was registered in the ISRCTN clinical trial database (registration number ISRCTN23993517).

### Participating centres

The participating centres were located in Germany and selected and coordinated by NETSTAP, which is a German, national network organisation for paediatricians conducting clinical research (Bochum, Germany).

### Subjects

Over a period of 23 months, full-term (≥37 weeks) infants whose mothers had decided not to exclusively breast-feed beyond the 34th day after birth were enrolled. Inclusion criteria were that infants had to be younger than age 35 d, exclusively formula-fed and of a normal birth weight for gestational age and sex (birth weight between the 10th and 90th percentile according to local standards on weight-for-gestational age values; Frank^(^^25^^)^). Exclusion criteria were allergic symptoms, i.e. atopic dermatitis and wheezing, antibiotics use prior to inclusion, congenital abnormality or chromosomal disorder that could affect normal growth, a parental history and pre- or perinatal indication for inherited immunodeficiency syndromes, or a congenital infection. *A priori*-defined protocol violations were defined as: (1) infants’ illness leading to violations of the protocol; (2) infants taking other foods/drinks than the study formula; water or sugar-free tea; (3) use of antibiotics during the intervention; (4) development of atopic dermatitis during the study according to the Hanifin criteria^(^^26^^)^; (5) deviation of the visit window (>3 d); (6) deviation in weight change >1 sd within the first 1–2 months of life according to infants’ individual target growth curve; (7) infant suffering from diarrhoea (≥3 liquid stools per d for >1 week); (8) >7 d of no declarable fever or sudden high fever without any demonstrable cause.

### Procedures

At enrolment, infants were randomly assigned to receive one of both formulas for a period of 13 weeks by four-block randomisation using research centre and family history of allergy as strata (at least one first-degree family member suffering from atopic eczema, rhinitis or asthma). Formulas were coded with letters (A, B, C, D) by the sponsor, and both the investigators and the infants’ parents were blinded to the formulas. For each centre a separate randomisation list with two strata was made. After randomisation by the investigator, parents received the assigned formula along with instructions for preparation. During the intervention period infants were fed *ad libitum* exclusively with their allocated formulas starting on the day of enrolment (aged 0–35 d) through to 13 weeks of intervention. The study consisted of a screening visit and four subsequent hospital visits at baseline, 4 weeks, 8 weeks, and 13 weeks of intervention. At each visit, parents received a diary for 4 weeks to record formula intake and tolerance on a daily basis. In this feeding record, the number of feedings and the amount of formula prepared and consumed per feeding were registered. Moreover, tolerance and stool characteristics were registered on a daily basis using a Likert scale^(^^27^^)^. In addition, parents were asked to record any medication, treatment or use of other nutrition during the intervention period. At the hospital visits, the physician monitored the growth of the infants and the presence of atopic symptoms of the skin and/or airways.

Three criteria were set as stopping rules for early termination of the study: (1) case of infection by *B. breve*; (2) >10 % of the subjects with deviating weight change (±1 sd) from individual target growth curve; (3) >10 % of the infants dropping out due to diarrhoea (≥3 liquid stools/d for more than 1 week).

As a quality-control procedure, an independent study monitor (CRO München) visited each study site prior to the study and regularly during the study to perform reviews of clinical research forms and subject diaries, as well as to check the investigational products and storage facilities.

### Study formulas

Both formulas were standard extensively hydrolysed (63 % of proteins <1000 Da) whey protein-based powder products with established anti-allergenic properties and intended to provide adequate nutritional support of infants in the first 6 months of life^(^^20^^,^^21^^)^. The formulas were isoenergetic and contained per 100 ml a similar amount of 66 kcal (276 kJ) energy, 1·6 g protein, 6·8 g carbohydrate, 3·6 g lipid, vitamins and minerals (Table 1). In contrast to the control formula, the synbiotic formula contained a specific mixture of short-chain galacto-oligosaccharides and long-chain fructo-oligosaccharides in a 9:1 ratio (0·8 g per 100 ml formula) and the probiotic strain *B. breve* M-16V (1·3 × 10^9^ colony-forming units per 100 ml formula; Morinaga Milk Industry Co., Ltd). The synbiotic and control formulas had a similar taste, smell and appearance and were supplied by Nutricia Research. Products were manufactured according to current good manufacturing practices, and were packaged and coded at Nutricia Research. The study products were stored at a cool, secure and limited access storage area protected from extremes of light, temperature and humidity.

**Table 1.** Composition of the two intervention products used in the study

Nutrient (per 100 ml)	Synbiotic	Control
Energy		
kcal	66	66
kJ	276	276
Protein (g; whey hydrolysate)	1·6	1·6
Fat (g)	3·6	3·6
Linoleic acid (g)	0·36	0·36
α-Linolenic acid (g)	0·07	0·07
Total carbohydrates (g)	6·8	6·8
Glucose (g)	0·1	0·1
Lactose (g)	2·6	2·6
Maltose (g)	0·5	0·5
Polysaccharides (g)	3·6	3·6
Transgalacto-oligosaccharides (g)	0·72	–
Inulin (g)	0·08	–
*Bifidobacterium breve* (cfu)	12·6 × 10^8^	–
Minerals		
Ca (mg)	52	52
P (mg)	26	26
Mg (mg)	5	5
Na (mg)	19	19
K (mg)	71	71
Cl (mg)	45	45
Fe (mg)	0·5	0·5
Zn (mg)	0·5	0·5
Cu (mg)	0·04	0·04
I (μg)	10	10
Se (μg)	1·2	1·2
Vitamins		
Vitamin A (μg RE)	60	60
β-Carotene (μg)	4·6	4·6
Vitamin D (μg)	1·5	1·5
Vitamin E (α-TE)	1·1	1·1
Vitamin K (mg)	5·2	5·2
Vitamin B_1_ (mg NE)	0·04	0·04
Vitamin B_2_ (mg)	0·1	0·1
Niacin (mg NE)	0·7	0·7
Vitamin B_6_ (mg)	0·04	0·04
Vitamin B_12_ (μg)	0·18	0·18
Vitamin C (mg)	8	8
Folic acid (μg)	11	11
Panthothenic acid (mg)	0·3	0·3
Choline (mg)	7	7
Taurine (mg)	4·6	4·6

cfu, Colony-forming units; RE, retinol equivalents; TE, tocopherol equivalents; NE, niacin equivalents.

### Measurements

The primary outcome of the study was defined as weight gain per d in infants fed the study formulas from age ≤35 to 119 d. The secondary outcomes were recumbent length, head circumference, symptoms of digestive tolerance, faecal microbiota and metabolic profile, plasma parameters of renal and liver function, presence of atopic symptoms and frequency of adverse events.

The infants were weighed naked while lying quietly on a calibrated electronic scale accurate to 10 g. Recumbent length was measured to the nearest 0·1 cm with a length board. Head circumference was measured using a non-stretch measuring tape. The investigators assessed the formula intake, the records of digestive tolerance and the adverse events at each visit. Average daily intake of the formula (ml/d) was calculated based on the parents’ monthly records. Infants were regarded as compliant if: (1) they had an average daily intake of >400 ml; (2) the average daily intake was higher than their age- and sex-specified minimum intake requirements; (3) the left-over, defined as prepared minus consumed, was below 25 %.

Symptoms of digestive tolerance consisted of daily stool frequency, stool consistency (on a five-point scale: 1 = watery, 2 = soft/pudding like, 3 = soft formed, 4 = dry formed, 5 = dry/hard pellets) and severity of constipation, diarrhoea, colic, vomiting, regurgitation, flatulence and nappy rash (0 = absent, 1 = mild, 2 = moderate, 3 = severe). In addition, crying frequency and sleeping behaviour were monitored (ten-point scale; rating of 10 for sleeping is very good, rating of 10 for crying is very often).

Any atopic dermatitis symptoms following clinical observation of the skin were recorded at the monthly visits using the SCORAD score^(^^28^^)^. Moreover, possible airway symptoms were documented. At the last visit (week 13), the SCORAD score of all infants was assessed.

The timing and duration, intensity and a description of the adverse events were documented by the parents in the daily diaries in between the study visits. An adverse event is considered any adverse change from baseline (pre-intervention) condition, which occurs during the course of the study after the intervention has started, whether considered related to the intervention or not. The investigators afterwards controlled the diaries and stated a possible relationship with the study product. A serious adverse event is defined as any untoward medical occurrence resulting in death, is life-threatening (at the time of the event), requires hospitalisation or prolongation of existing hospitalisation or results in persistent or significantly disability or incapacity. Investigators were obliged to report a serious adverse event within 24 h of occurrence to the medical monitor and sponsor of the study. Faecal and blood samples were collected at four of the seventeen sites. Blood samples (twenty-five for synbiotic and forty-one for control) were collected at baseline and at the end of intervention (week 13) to determine blood parameters indicative for renal and liver functioning consisting of: number of leucocytes; thrombocytes; lymphocytes; monocytes; eosinophils; basophils and erythrocytes; Hb (g/l); haematocrit (%); mean corpuscular volume (fl); mean corpuscular Hb (pg); mean corpuscular Hb concentration (g/l); segmented neutrophils (%); neutrophils (%); total protein (g/l); albumin (g/l); urea (%); creatinine (mg%); alanine aminotransferase (ALAT, GPT; U/l); aspartate aminotransferase (ASAT, GOT; U/l); γ-glutamyl transpeptidase (U/l); and pre-albumin (mg/l).

Faecal samples (twenty-four and thirty-six infants in the synbiotic group *v*. control group) were collected at baseline (before start of the intervention), week 1 and week 13 of the intervention. Directly after collection two tubes with stool sample were stored at −20°C by the parents and transported to the hospital in a cold storage bag as soon as possible. The microbial composition of the faecal samples was determined by fluorescent *in situ* hybridisation (FISH) using specific 16S rRNA-targeted oligonucleotide probes specific for these bacteria groups^(^^19^^,^^29^^)^ (bifidobacteria, lactobacilli, *Bacteroides/Prevotella, Clostridium histolyticum/C. lituseburense*, Enterobacteriaceae and *C. coccoides/Eubacterium rectale*). The nucleic acid stain DAPI (4',6-diamidino-2-phenylindole) (Invitrogen) was used for determining total faecal cell counts. Paraformaldehyde-fixed faecal samples were hybridised with the specific probes as described previously^(^^30^^)^. Thereafter the samples were counted using an automated Olympus AX70 epifluorescence microscope equipped with a Lang LStep13 8 slides-stage (Paes Nederland bv) and an F-View II charge-coupled device (Soft Imaging System GmbH) and image analysis software. The percentage of bacterial cells was determined at twenty-five randomly chosen positions on each well by counting all cells using a DAPI filter set (SP100; Chroma Technology Corp.) and by counting the targeted bacterial group using a Cy3 filter set (41007; Chroma Technology Corp.).

In addition, pH, concentrations of SCFA and lactate were measured in the faecal samples (as described in Knol *et al.*^(^^31^^)^).

### Statistics

All statistical analyses were performed using SAS 9.2 (SAS Institute, Inc.). Primary outcome was the difference in weight gain per d during 13 weeks of intervention with the objective to demonstrate growth equivalence. Growth of infants in the synbiotic and control groups was considered equivalent when the two-sided 90 % CI of the difference in the means of weight gain laid within the interval from −0·5 to +0·5 sd^(^^32^^)^. This is equivalent to the two one-sided testing approach. The required sample size for two one-sided statistical testing using *α* = 0·05 in each and a power = 0·80 was sixty-nine infants per group. Taking a drop-out rate of 25 % into account, a total of 152 had to be enrolled. An ANCOVA method was used in the equivalence analysis, taking study centre, risk for allergy, sex, and weight at baseline as covariates. In the analysis, centres that included ≤4 infants at week 13 in the growth analysis were pooled to one centre.

Secondary outcomes included calculated anthropometric *z*-scores using WHO growth standards as reference^(^^33^^)^. All anthropometric results will be given for both per-protocol (PP) and intention-to-treat (ITT) populations; the remaining parameters will mainly be described for the ITT population. For evaluating the difference from the WHO growth standards, a repeated-measures mixed model (RMMM) was used per group with a random intercept and slope for subjects and age as the fixed effect. Parameters consisting of continuous data were analysed using the two-sample *t* test, or, in the case of non-normality (evaluated by the Shapiro–Wilk test with *α* = 0·05), the Mann–Whitney test (comparing means) or the Jonckheere–Terpstra test (ordinal data). Categorical parameters were analysed using Fisher's exact test. With respect to secondary parameters, blood and stool samples were only collected in a subgroup of subjects recruited at four of the seventeen sites. Power calculations based on differences in albumin levels, assuming 1 sd as a clinically relevant difference and using *α* = 0·05 and a power = 0·80, indicated a total of sixteen infants per group was required. Assuming a drop-out rate of 20 %, a total of forty infants needed to be included in the subpopulation to meet the required amount of infants per arm. During the study it became evident that for some of the faecal samples the collected aliquots did not contain enough faeces to perform the planned analyses. Moreover, for some infants, the stool samples were not collected. Hence, sample collection at these sites was continued to secure a sufficient amount of samples for the stool sample analysis.

## Results

### Subject characteristics

**Fig. 1.**
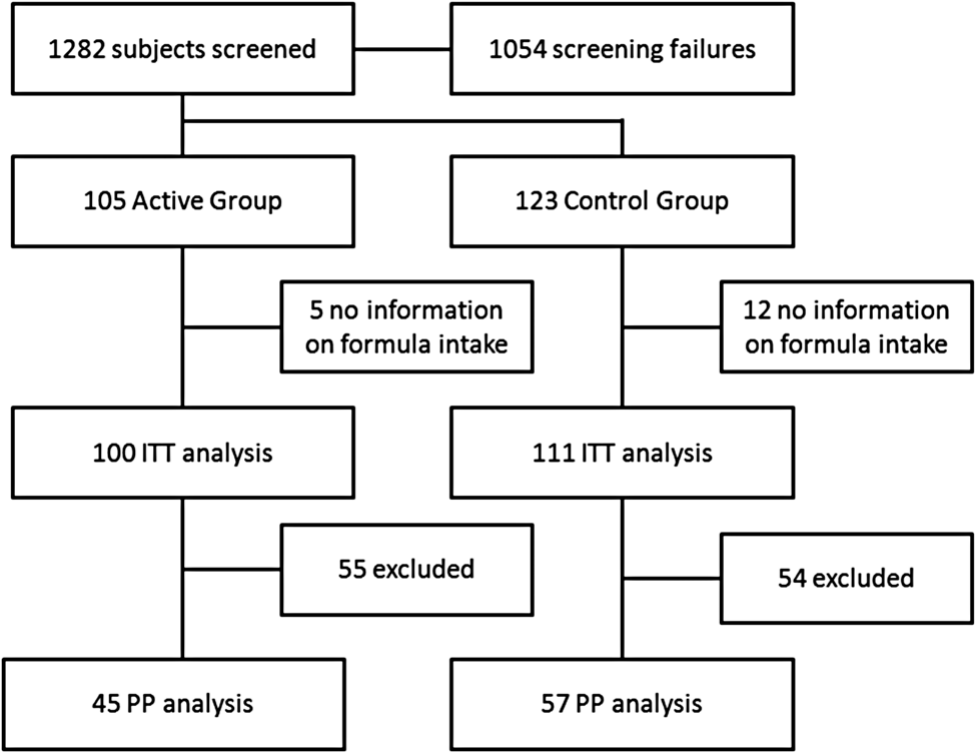
Disposition of study subjects. A total of forty-six infants were excluded from the per-protocol (PP) group due to major protocol deviations; a total of sixty-three infants dropped-out during the study. ITT, intention to treat.

From April 2005 to November 2006 a total of 1282 subjects were screened, of which 228 subjects were randomised to one of the infant formulas. A flowchart showing the enrolment and disposition of the study subjects is given in Fig. 1. The large number of screening failures was due to the fact that most of the infants were not exclusively formula fed at the time of screening. Of the 228 infants that were recruited, 211 infants received at least some of the study formula and constituted the ITT study population, excluding seventeen infants from further analyses (twelve in the control group and five in the synbiotic group). A substantial amount of 109 infants was excluded from the PP population (fifty-four for the control group and fifty-five for the synbiotic group) due to protocol violations or dropping-out from the study. In total, forty-six subjects were recorded as having major protocol violations and were excluded from the PP groups. The protocol violations included erroneous enrolment (not meeting criteria; *n* 17), use of probiotics during the study (*n* 5), non-compliance (*n* 10) and/or consumption of mislabelled products (*n* 15). In addition, a total number of sixty-three infants dropped out during the study. The reasons for dropping out did not show a significant difference between the groups (Table 2). With regards to demographics of the ITT population, infants of the synbiotic group were more often male (Fisher's exact test; *P* = 0·027), had a higher gestational age and weight at birth (Mann–Whitney test, *P* = 0·035; *t* test, *P* = 0·012) and a higher length, but not weight, at baseline (Mann–Whitney test; *P* = 0·046) and had a higher prevalence of atopic skin symptoms at baseline (Fisher's exact test; *P* = 0·008) compared with infants of the control group (Table 3). In the PP population, only the higher prevalence of atopic skin symptoms remained significant (8 *v*. 0 % for synbiotic *v*. control; Mann–Whitney test, *P* = 0·042). However, maternal weight was higher (75 (sd 16·5) *v*. 66·9 (sd 12·5) kg; *t* test, *P* = 0·006) and maternal height tended to be higher (1·70 (sd 0·06) *v*. 1·67 (sd 0·06) m; *t* test, *P* = 0·067)) in the synbiotic group compared with the control group, ultimately resulting in a higher maternal BMI in the synbiotic group (26·04 (sd 5·38) kg/m^2^) compared with the control group (23·9 (sd 4·02) kg/m^2^; *t* test *P* = 0·027)

**Table 2.** Drop-out number and reasons during the study*

Reason for drop-out	Synbiotic (*n*)	Control (*n*)
Use of antibiotics	5	4
Hospitalisation	0	1
Refusal of formula	5	6
Unsatiated	4	5
Atopic dermatitis	2	0
Consent withdrawal	1	2
Crying	0	2
Constipation	4	5
Diarrhoea	2	1
Vomiting	2	9
Abdominal pain	1	2
Other reasons	1	2

* Fisher's exact test was used to evaluate potential differences between the treatment groups.

**Table 3.** Birth and baseline characteristics of subjects in the intention-to-treat population (Mean values and standard deviations, or percentage)

	Synbiotic (*n* 100)		Control (*n* 111)	
	Mean	sd	Mean	sd
Birth weight (g)‡	3450*	434	3300	424
Gestational age (weeks)§	39·4*	1·2	39·0	1·4
Vaginal delivery (%)‖	75·8		67·6	
Sex (% male)‖	59·0*		43·2	
Weight at baseline (g)‡	4031	665	3892	685
Length at baseline (cm)‡	53·2*	2·2	52·4	2·6
Head circumference at baseline (cm)‡	36·6†	1·6	36·2	1·6
Age at baseline (d)§	23·3	11·7	22·5	11·2
Maternal height (m)‡	1·67	0·07	1·67	0·06
Maternal weight (kg)§	74·4	17·6	70·4	15·7
Maternal BMI (kg/m^2^)‡	26·5†	5·6	25·2	5·4
Paternal height (m)‡	1·80	0·08	1·80	0·08
Paternal weight (kg)§	81·4	13·1	83·5	13·4
At risk for atopy (%)‖	39·0		42·3	
Atopic skin symptoms (% infants)‖	11·0*		1·8	

* Value was significantly different from that for the control group (*P* < 0·05).

† Value tended to be significantly different from that for the control group (0·10 > *P* > 0·05).

‡ Two-sample *t* test.

§ Mann–Whitney test.

‖ Fisher's exact test.

### Formula consumption

The observed formula intake was in line with expectations, and infants of the synbiotic and control groups consumed similar, increasing amounts of formula in the first, second and third month after the start of the intervention (Table 4; *t* test *P* = 0·973, *P* = 0·475 and *P* = 0·852, respectively).

**Table 4.** Daily formula intake of the synbiotic and control groups in the per-protocol population* (Mean values and standard deviations)

	Synbiotic (*n* 45)		Control (*n* 57)	
Formula intake (ml/d)	Mean	sd	Mean	sd
Baseline – week 4	672	108	689	111
Week 4 – week 8	758	110	758	140
Week 8 – week 13	796	101	795	100

* Statistical comparisons were performed using a two-sample *t* test.

### Infant growth patterns

**Fig. 2.**
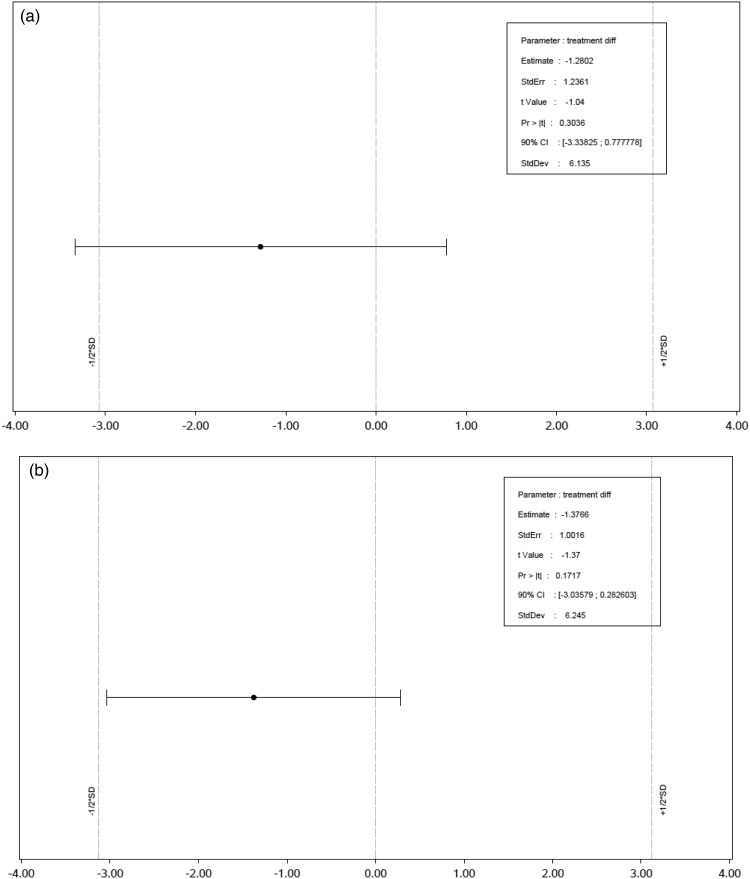
Equivalence testing for weight gain (g/d) during the intervention period in the per-protocol population (a) and the intention to treat population (b). An ANCOVA method was used in the equivalence analysis, taking study centre, risk for allergy, sex, and weight at baseline as covariates.

Average weight gain (g/d) during the study period was not statistically different between the synbiotic and control groups (Table 5). Equivalence in weight gain per d could not be demonstrated for the PP population (Fig. 2). The 90 % CI of the difference in means of weight gain slightly crossed the lower 0·5 sd margin (ANCOVA, 90 % CI (−3·3 to +0·8 g/d) and the margin ±3·1 g/d, respectively). However, in the ITT population the equivalence in weight gain per d was demonstrated, with an 90 % CI of the difference in means that lay well within the equivalence margins (ANCOVA, 90 % CI (−3·0 to +0·3 g/d) and the margin ±3·1 g/d, respectively) despite an even slightly larger point estimate of the difference in means compared with the PP population (Fig. 2). Inclusion of maternal BMI as a covariate did not affect the outcome of the equivalence analyses in the PP or ITT population (data not shown). At all visits during the intervention period, the mean body weight of infants in the synbiotic and control groups were similar both for the ITT and the PP populations (ANCOVA, *P* > 0·10; data not shown). The overall weight-for-age development during the intervention period was in line with the sex-specific WHO child growth standard, which represents the growth of exclusively breast-fed infants. As depicted in Table 6, weight-for-age *z*-scores of infants in both groups of the PP population were very close to zero (RMMM *P* = 0·130 and *P* = 0·102 for the control and synbiotic groups). In contrast to the synbiotic group, infants of the control group had an increase in weight-for-age *z*-score over time, resulting in a significantly different slope compared with the WHO standard (RMMM, effect for age, *P* = 0·572 and *P* = 0·002, respectively).

**Table 5.** Weight gain, length gain and head circumference gain during the intervention period* (Mean values and standard deviations)

	ITT population				PP population			
Intervention group…	Synbiotic (*n* 100)		Control (*n* 111)		Synbiotic (*n* 45)		Control (*n* 57)	
	Mean	sd	Mean	sd	Mean	sd	Mean	sd
Weight gain (g/d)	27·5	0·7	28·5	0·7	28·7	6·4	29·8	6·0
Length gain (cm/week)	0·77†	0·02	0·81	0·02	0·77	0·13	0·82	0·16
Head circumference gain (cm/week)	0·36	0·01	0·38	0·01	0·37	0·01	0·39	0·01

ITT, intention-to-treat; PP, per-protocol.

* The ANCOVA method was used to evaluate differences taking study centre, risk for allergy, sex, and weight at baseline as covariates.

† Tendency for a lower mean length gain compared with control (*P* = 0·093).

**Table 6.** WHO weight-for-age *z*-scores and length-for-age *z*-scores in the intention-to-treat (ITT) and per-protocol (PP) populations (Mean values and standard deviations)

	ITT population				PP population			
Intervention group…	Synbiotic (*n* 100)		Control (*n* 111)		Synbiotic (*n* 45)		Control (*n* 57)	
	Mean	sd	Mean	sd	Mean	sd	Mean	sd
Weight-for-age *z*-scores								
Baseline	−0·101	0·854	−0·253	0·896	−0·161	0·729	−0·224	0·905
Week 4	−0·148	0·778	−0·270	0·957	−0·178	0·733	−0·175	0·910
Week 8	−0·171	0·824	−0·204	0·950	−0·212	0·851	−0·164	0·965
Week 13	−0·060	0·824	0·051	1·012	0·100	0·844	0·042	1·006
Length-for-age *z*-scores								
Baseline	−0·030	0·948	−0·285	1·033	−0·071	0·869	−0·375	1·138
Week 4	−0·035	1·000	−0·206	1·054	−0·073	0·869	−0·172	1·089
Week 8	0·041	0·985	−0·044	1·099	0·026	0·905	0·045	1·148
Week 13	0·235	1·025	0·321	1·259	0·228	1·031	0·248	1·266

In the ITT population, no significant differences in length or length gain between the two groups were observed. In the PP population, the mean length gain was not different between the synbiotic compared with the control group (Table 5; ANCOVA, *P* = 0·11), although a lower absolute length was observed in the synbiotic *v*. the control group at week 4 (56·0 (sd 0·2) *v*. 56·7 (sd 0·2) cm; ANCOVA, *P* = 0·031), week 8 (59·2 (sd 0·2) *v*. 60·1 (sd 0·2) cm; ANCOVA, *P* = 0·003), and week 13 (62·5 (sd 0·3) *v*. 63·4 (sd 0·3) cm; ANCOVA, *P* = 0·022). During the intervention period the length-for–age *z*-scores using the sex-specific WHO child standard were observed to be close to zero in both groups of the PP population (Table 6; RMMM *P* = 0·331 and *P* = 0·868 for the control and synbiotic groups, respectively). The mean head circumference gain, as well as the head circumference at each visit (data not shown) were not significantly different between the synbiotic and control groups in both the ITT and PP populations (ANCOVA; *P* > 0·10 for all; Table 5).

### Adverse events and blood parameters

The percentage of children with at least one (serious) adverse event during the study was similar between treatment groups (15·0 *v*. 19·8 % for synbiotic and control groups; Fisher's exact test, *P* = 0·372). Except for non-infection-related respiratory tract diseases or symptoms, which were higher in the synbiotic compared with the control group of the ITT population (8·0 *v*. 1·8 %; Fisher's exact test, *P* = 0·049), the frequency of specific diseases related to the adverse events were not significantly different between treatment groups (data not shown).

One infant developed atopic dermatitis which was reflected in a higher eosinophil count after 13 weeks of intervention in the synbiotic group of the ITT subpopulation (median 4·2 *v*. 2·9 %; Mann–Whitney test, *P* = 0·029); omitting this value resulted in similar counts compared with the control group. No differences in any of the other blood parameters were observed compared with the control group (data not shown).

### Gastrointestinal tolerance

With respect to tolerance data, only the stool consistency score in the first 4 weeks of the intervention period was lower in the synbiotic *v*. control group of the ITT subpopulation (Jonckheere–Terpstra test; *P* = 0·035), which was accompanied by a lower severity of nappy rash in the synbiotic group in that particular period (Jonckheere–Terpstra test; *P* = 0·026; Table 7). At the end of the intervention period, stool frequency tended to be lower in the synbiotic group *v*. control group of the ITT subpopulation (Mann–Whitney test, *P* = 0·056; Table 7).

**Table 7.** Tolerance parameters of infants in the synbiotic and control groups in the intention-to-treat population (Mean values and standard deviations, and number of infants)

		Synbiotic			Control		
Parameter	Time (weeks)	Mean	sd	*n*	Mean	sd	*n*
Stool frequency (*n*/d)	0–4	1·4	0·97	95	1·5	0·93	105
	4–8	1·2	0·72	77	1·2	0·65	79
	8–13	1·1†	0·59	74	1·2	0·56	74
Stool consistency score‡	0–4	2·2*	0·8	95	2·3	0·7	105
	4–8	2·1	0·5	77	2·2	0·6	79
	8–13	2·1	0·5	74	2·2	0·6	74
Constipation severity§	0–4	0·1	0·5	89	0·2	0·6	103
	4–8	0·0	0·2	71	0·0	0·1	76
	8–13	0·0	0·0	68	0·0	0·1	71
Diarrhoea severity	0–4	0·1	0·3	89	0·1	0·3	101
	4–8	0·0	0·2	71	0·0	0·1	76
	8–13	0·0	0·1	68	0·0	0·1	71
Colic severity	0–4	0·2	0·6	89	0·2	0·6	103
	4–8	0·1	0·4	72	0·2	0·6	79
	8–13	0·0	0·2	68	0·0	0·2	72
Vomiting severity	0–4	0·1	0·3	90	0·2	0·6	103
	4–8	0·1	0·3	72	0·1	0·3	77
	8–13	0·1	0·2	68	0·0	0·2	71
Regurgitation severity	0–4	0·8	0·8	91	0·8	0·7	106
	4–8	0·7	0·7	74	0·7	0·7	79
	8–13	0·7	0·7	71	0·7	0·8	74
Flatulence severity	0–4	0·9	0·9	91	0·8	0·8	105
	4–8	0·8	0·8	75	0·7	0·8	79
	8–13	0·6	0·8	71	0·5	0·6	74
Nappy rash severity	0–4	0·0	0·3	88	0·1	0·3	103
	4–8	0·1	0·3	71	0·1	0·2	77
	8–13	0·1	0·3	68	0·1	0·3	73
Crying severity‖	0–13	4·1	1·9	93	4·1	2·1	101
Sleeping score‖	0–13	7·2	1·9	94	7·1	2·0	101

* Mean value was significantly different from that for the control group (*P* < 0·05).

† Mean value tended to be significantly different from that for the control group (0·10 > *P* > 0·05).

‡ Consistency scores: 1 = watery; 2 = soft/pudding like; 3 = soft formed; 4 = dry formed; 5 = dry/hard pellets.

§ Severity scores: 0 = absent; 1 = mild; 2 = moderate; 3 = severe.

‖ Crying frequency and sleeping scores were measured on a ten-point scale: a rating of 10 for sleeping is very good; a rating of 10 for crying is very often. Stool consistency and gastrointestinal symptoms comparisons were tested using the Jonckheere–Terpstra test, stool frequency comparisons were tested using the Mann–Whitney test.

### Faecal microbiota, SCFA and lactate

Faecal samples of a subpopulation of sixty infants (twenty-four for the synbiotic group and thirty-six for the control group) were to be analysed at baseline, at week 1 and at 13 weeks after intervention (a total of 180 samples). In total, twenty-seven samples were missing and another fourteen samples did not contain a sufficient amount of faecal matter to perform all planned analysis, resulting in a somewhat lower number for some time points. At baseline, faecal microbiota and related stool parameters were not significantly different between the synbiotic *v*. control group of the ITT subpopulation (Tables 8 and 9).

**Table 8.** Faecal microbiota composition (%) over time in a subgroup of infants in the synbiotic and control groups of the intention-to-treat population (Medians, minimum–maximum and number of infants)

		Synbiotic			Control		
Microbiota	Time	Median	Range	*n*	Median	Range	*n*
*Bacteroides*/*Prevotella* group	Baseline	10^−4^	10^−4^–5·6	21	10^−4^	10^−4^–6·4	32
	Week 1	10^−4^	10^−4^–0·8	22	10^−4^	10^−4^–6·8	30
	Week 13	10^−4^	10^−4^–10^−4^	19	10^−4^	10^−4^–7·4	27
Bifidobacteria	Baseline	56·4	10^−4^–88·8	21	48·5	10^−4^–77·3	32
	Week 1	58·6†	10^−4^–81·0	21	42·6	5·7–79·7	29
	Week 13	60·1*	27·0–89·4	19	48·3	9·3–76·0	27
*Clostridium histolyticum/C. lituseburense* group	Baseline	10^−4^	10^−4^–19·8	21	10^−4^	10^−4^–21·0	32
	Week 1	10^−4^*	10^−4^–5·5	22	2·8	10^−4^–21·5	30
	Week 13	0·2*	10^−4^–11·5	19	2·6	10^−4^–29·2	27
Enterobacteriaceae	Baseline	10^−4^	10^−4^–24·9	21	0·2	10^−4^–18·5	31
	Week 1	10^−4^	10^−4^–43·6	21	2·5	10^−4^–21·8	28
	Week 13	10^−4^	10^−4^–10·6	19	0·2	10^−4^–10·6	27
*C. coccoides/Eubacterium rectale* group	Baseline	10^−4^†	10^−4^–17·3	20	10^−4^	10^−4^–46·2	32
	Week 1	10^−4^	10^−4^–16·3	22	10^−4^	10^−4^–29·0	30
	Week 13	1·9†	10^−4^–26·3	19	13·7	10^−4^–43·3	26
Lactobacilli/enterococci	Baseline	0·3	10^−4^–24·0	20	0·5	10^−4^–20·2	32
	Week 1	0·1	10^−4^–11·6	22	1·1	10^−4^–17·9	30
	Week 13	0·2	10^−4^–10·6	19	0·1	10^−4^–12·8	26

* Median value was significantly different from that for the control group (*P* < 0·05; Mann–Whitney test).

† Median value tended to be significantly different from that for the control group (0·10 > *P* > 0·05; Mann–Whitney test).

**Table 9.** SCFA, branched-chain fatty acids, lactate (mmol/kg wet weight) and pH in the stools of a subgroup of infants in the intention-to-treat population (Medians, minimum–maximum and number of infants)

		Synbiotic			Control		
Parameter	Time	Median	Range	*n*	Median	Range	*n*
Acetic acid	Baseline	48·50	0–143	20	44·20	0–115	32
	Week 1	34·00	12–95	21	39·11	0–74	28
	Week 13	38·50	18–59	16	46·06	18–144	25
Butyric acid	Baseline	0·46	0·00–10·73	20	0·00	0·00–8·31	32
	Week 1	0·00	0·00–3·56	21	1·08	0·00–12·44	28
	Week 13	3·36	0·00–8·95	16	3·08	0·00–15·09	25
Propionic acid	Baseline	8·78	0·00–36·20	20	8·25	0·00–24·38	32
	Week 1	6·10†	1·49–11·80	21	8·80	0·00–48·20	28
	Week 13	10·64	1·99–23·10	16	9·70	2·10–21·56	25
Valeric acid	Baseline	0·00	0·00–0·00	20	0·00	0·00–0·00	32
	Week 1	0·00	0·00–0·93	21	0·00	0·00–1·40	28
	Week 13	0·00	0·00–0·88	16	0·00	0·00–1·79	25
d-Lactate	Baseline	0·68	0·00–4·92	21	0·60	0·00–6·57	33
	Week 1	0·06	0·00–1·65	22	0·11	0·00–1·95	29
	Week 13	0·00*	0·00–1·02	19	0·00	0·00–0·48	27
l-Lactate	Baseline	0·47	0·00–14·00	21	0·00	0·00–6·31	33
	Week 1	1·12	0·00–4·25	22	0·60	0·00–11·38	29
	Week 13	0·58	0·00–3·34	19	0·75	0·00–4·14	27
Isobutyric acid	Baseline	0·00	0·00–2·10	20	0·00	0·00–1·50	32
	Week 1	0·00*	0·00–1·10	21	0·76	0·00–2·40	28
	Week 13	0·00†	0·00–1·90	16	0·88	0·00–2·76	25
Isovaleric acid	Baseline	0·00	0·00–2·60	20	0·00	0·00–2·92	32
	Week 1	0·00*	0·00–1·93	21	1·00	0·00–5·35	28
	Week 13	0·85	0·00–2·69	16	1·41	0·00–5·25	25
pH	Baseline	5·98	5·17–7·79	21	6·14	5·08–7·72	34
	Week 1	6·02*	5·23–8·28	23	6·32	4·95–7·48	30
	Week 13	5·92†	5·23–8·01	20	6·39	5·46–7·21	27

* Median value was significantly different from that for the control group (*P* < 0·05; Mann–Whitney test).

† Median value tended to be significantly different from that for the control group (0·10 > *P* > 0·05; Mann–Whitney test).

A statistically significantly higher level of the bifidobacteria population was observed at week 13 in the synbiotic compared with the control group (Mann–Whitney test, *P* = 0·014), whereas only a trend towards a higher level was found at week 1 in the synbiotic group. A significantly lower percentage of the potential pathogens represented by the *C. histolyticum/C. lituseburense* cluster was observed in the synbiotic *v*. the control group at both intervention time points (Mann–Whitney test, *P* = 0·003; and Mann–Whitney test, *P* = 0·013, respectively). In addition, the level of *C. coccoides/E. rectale* cluster tended to be lower in the synbiotic group at week 13 compared with the control group (Mann–Whitney test, *P* = 0·052).

No differences in faecal acetate or butyrate levels were observed during the intervention period. Compared with the control group, faecal propionic acid levels tended to be lower after 1 week of intervention in the synbiotic group (Mann–Whitney test, *P* = 0·089). The concentrations of the branched SCFA such as isobutyrate and isovalerate were statistically significantly lower in the synbiotic group compared with the control group following 1 week of intervention (Mann–Whitney test, *P* = 0·002; and Mann–Whitney test, *P* = 0·003, respectively). No differences were observed in l-lactate levels and although the d-lactate levels were low, a slightly higher concentration of d-lactate was observed at week 13 in the synbiotic group (Mann–Whitney test; *P* = 0·04). Furthermore, supplementation with the synbiotic formula resulted in a statistically significantly lower faecal pH from week 1 in the synbiotic group than in the control group and only a trend was observed at week 13 (week 1: Mann–Whitney test, *P* = 0·044; week 13: Mann–Whitney test, *P* = 0·062).

The results of the PP population (data not shown) confirmed the above results observed in the ITT population and showed a stronger effect of the synbiotic intervention. Indeed, the percentage of bifidobacteria in the synbiotic group was statistically significantly higher than in the control group from the first week of the intervention (Mann–Whitney test, *P* = 0·04) and this effect was maintained until the end of the intervention. A significantly lower percentage of the potential pathogens represented by the *C. histolyticum/C. lituseburense* cluster was observed in the synbiotic *v*. the control group at both intervention time points (week 1: Mann–Whitney test, *P* = 0·043; week 13: Mann–Whitney test, *P* = 0·058). Faecal pH was also statistically significantly lower in the synbiotic compared with the control group at week 1 (Mann–Whitney test; *P* = 0·007) and week 13 (Mann–Whitney test; *P* = 0·021). At the end of the intervention, the *C. coccoides/E .rectale* cluster was statistically significantly lower in the synbiotic than in the control group (Mann–Whitney test; *P* = 0·013).

### Atopic symptoms

At baseline, the prevalence of atopic symptoms, especially atopic skin symptoms, was higher in the synbiotic group compared with the control group of the ITT population (12·0 and 2·7 % of the infants, respectively; Fisher's exact test, *P* = 0·013). During and at the end of the study, no differences were statistically significantly confirmed in atopic symptoms between the two groups (data not shown).

## Discussion

This is the first longitudinal study to evaluate the effect of an extensively hydrolysed formula with the specific combination of the prebiotic mixture scGOS/lcFOS (9:1) and the probiotic strain *B. breve* M-16V on growth and tolerance in healthy, term infants. Given predefined equivalence margins, weight gain per d following 13 weeks of formula feeding was proven to be equivalent between the synbiotic group and control group in the ITT population, but not in the PP population. However, the weight-for-age *z*-scores based on WHO growth standards of infants in the two groups of the PP population were close to zero during the entire intervention period. Absolute length gain was slightly decreased in the synbiotic population, but the length-for-age *z*-score values were close to zero according to WHO growth standards throughout the study period for both the synbiotic group and control group. Additional safety and tolerance parameters like head circumference, gastrointestinal parameters, number and type of adverse events, blood parameters indicative for renal or liver function, gastrointestinal parameters, and atopic symptoms were similar between the two groups following 13 weeks of intervention.

The weight gain per d during 13 weeks of intervention was similar in both groups in the PP population. Since the 90 % CI of the difference estimate slightly crossed the predefined equivalence margin of −0·5 to +0·5 sd, equivalence in weight gain during 13 weeks of intervention was not demonstrated between groups in the PP population. However, the point estimate of the difference in the PP population was smaller compared with that observed in the ITT population in which equivalence in growth was demonstrated. Hence, the main reason for the inability to demonstrate equivalence is most probably the lack of power in the PP population due to a much higher than expected drop-out rate. To safeguard 80 % power for statistical evaluation of weight gain, a minimum of sixty-nine infants per treatment group was estimated as the required sample size, but the synbiotic group and control group of the PP population only consisted of forty-five and fifty-seven infants, respectively. Moreover, weight-for-age z-scores indicated growth rates not different from WHO growth standards. Since these standards are based on the growth curve of exclusively breastfed infants, which is universally considered to be the most optimal nutrition for infants, this is indicative of a sufficient nutritional efficacy of both formulas.

As described in a systematic review by Mugambi *et al*. a limited number of publications previously described the impact of different combinations of pre- and probiotics on infant growth and safety^(^^34^^–^^37^^)^. Although investigating different mixtures of pre- and probiotics (in a different nutritional composition), all three studies concluded that the use of those specific mixtures is safe, well-tolerated and supports adequate growth in healthy, term infants. It should be noted, though, that the predefined equivalence margins for weight gain during 3 months of intervention in these studies are considerably wider ranging from ±3·9 to ±5·4 g/d compared with that applied in our study (±3·1 g/d). Interestingly, if we would have applied a similar (wider) equivalence margin in the present study, we would have demonstrated equivalence in weight gain per d for the PP population in this study as well. Although previous studies have demonstrated adequate growth of healthy term infants on formulas containing various mixtures of pre- and probiotics, caution should be used drawing conclusions on the safety of these mixtures in general, since different mixtures can have different physiological effects. Moreover, ‘negative’ results are less likely to be published and some clinical studies might even have been stopped prematurely. The secondary outcome measures absolute length and length gain were lower in the synbiotic group compared with the control group in the PP population, but not in the ITT population, during the intervention period. This is in contrast to the pre- and probiotics studies mentioned previously that indicated a similar length and/or length gain during the intervention periods^(^^34^^–^^37^^)^. However, the length-for-age *z*-scores of the synbiotic group in this study were not different from the WHO growth standard for both the ITT and PP populations, indicating an adequate growth in height.

The percentage of infants with at least one (serious) adverse event was similar between treatments groups (15·0 and 19·8 % for the synbiotic group and control group). The type of adverse events reported is typical for young infants and no relevant difference in type of adverse events was observed between treatment groups. Moreover, the addition of the pre- and probiotic mixture did not affect formula consumption (ml/d), indicating a good acceptance and palatability. Infants fed the experimental formula had slightly softer stools in the first 4 weeks of the intervention, which is in line with previously reported stool-softening effects of this specific prebiotic mixture^(^^38^^)^. This is considered to be beneficial, since breast-fed infants have, in general, a higher stool frequency and softer stools compared with cows’ milk-based formula-fed infants^(^^39^^)^.

After 13 weeks of the intervention, supplementation with scGOS/lcFOS (9:1) and *B. breve* M-16V in the synbiotic group resulted in a higher percentage of bifidobacteria (60 *v*. 48 %), a lower percentage of potential pathogens such as the clostridia-related species, represented by *C. lituseburense/C. histolyticum* (0·2 *v*. 2·6 %) and a tendency for a lower percentage of the *C. coccoides/E. rectale* (1·9 *v*. 13·7 %) cluster compared with the control group (ITT population). These findings are in line with previous observations from an intervention study using this synbiotic mixture in older infants with atopic dermatitis as well as from an intervention study using a formula with scGOS/lcFOS (9:1) only in 2-month-old infants^(^^15^^,^^27^^)^. Since the number of children in the synbiotic group and control group receiving breast milk before the start of the intervention was similar (13 *v*. 10 %, respectively) as well as their baseline values, the observed effect can be fully attributed to the supplementation of scGOS/lcFOS and *B. breve* M-16V in the formula. As demonstrated in a previous study with this synbiotic concept^(^^18^^)^, the induced shift in microbiota composition was accompanied by changes in faecal metabolic profile, evident from the reduced levels of faecal pH and the branched SCFA isobutyric and isovaleric acids, which might be an indication of decreased proteolytic fermentation^(^^40^^)^. Although we did not demonstrate a statistically significant difference in the other SCFA (acetate, propionate and butyrate), the levels of the other SCFA (acetate, propionate and butyrate) were lower than those observed in the control group at the end of the intervention, although no statistically significant differences were demonstrated. This is most probably due to the high inter-individual variability observed between subjects. It is anticipated that these changes might have a beneficial impact on the priming of the immune system, potentially strengthening the effects of preventive treatment by reducing the risk of development of allergic diseases^(^^19^^)^. Furthermore, recent evidence suggests that early-life microbiota colonisation plays a crucial role in the host metabolic pathways and an early gut microbiota perturbation due to antibiotic use and the subsequent aberrant metabolic maturation are believed to be an important element in the development of obesity in later life^(^^41^^,^^42^^)^. Given the lack of long-term follow-up due to the safety design of this study, it is, however, beyond the scope of this paper to speculate on the potential effects of the observed changes in microbiota and faecal metabolic profile associated with the synbiotic concept on growth and body composition development.

Hydrolysed formulas are intended for use in infants with family history of allergy or suffering from allergic diseases to prevent or alleviate allergic symptoms. This study in healthy, term infants evaluates the safety of the addition of a specific synbiotic concept to an extensively hydrolysed formula with demonstrated nutritional adequacy for healthy and allergic infants^(^^20^^,^^43^^)^. It has been shown that irrespective of type of feeding, infants with atopic dermatitis have a reduced growth during infancy starting already from the first months of life^(^^44^^)^. In the present study, the presence of atopic dermatitis at the screening visit or its occurrence during the study was a predefined exclusion criterion. In addition, the presence of family history for allergy was recorded at the screening visit and used as a stratification factor. Using family history of allergy as stratification was anticipated to even the risk for potential drop-out due to the development of atopic dermatitis between the synbiotic group (*n* 2) and the control group (*n* 0). More importantly, we anticipated that it would increase the likelihood to detect a potential effect of the synbiotic concept on the microbial parameters or development of atopic symptoms.

Some limitations of our study need to be addressed. Some of the baseline characteristics were different between the two intervention arms of the ITT population, including a higher birth weight, higher gestational age, more male infants and higher length and head circumference at baseline for the infants in the synbiotic group compared with the control group. In order to minimise any bias related to these differences on the primary outcomes, sex and weight at baseline were included as covariates in the equivalence analysis. In the PP population, none of these factors was significantly different between the intervention groups, which could have been the result of the low number of infants in this population. Maternal weight was significantly higher and maternal height tended to be for the synbiotic group compared with the control group of the PP population, but inclusion of maternal BMI as a covariate did not affect the outcome of the equivalence analyses in the PP or ITT population. The drop-out rate of 33 % in our study was at the high end of the range in drop-out rate between 20 to 33 % reported in similar studies^(^^34^^–^^36^^)^. One of the potential reasons for this relatively high drop-out rate could have been the lower palatability of hydrolysed infant formulas compared with standard infant milk formulas^(^^45^^)^, which might have been the underlying factor for a substantial amount of drop-outs (20/63 due to formula refusal or an unsatiated infant). The drop-out rates as well as the reported reasons for drop-out were not different between treatment groups, indicating that it is not associated with the presence of the pre- and probiotic mixture in the experimental formula. The drop-out rate and number of subjects that were recorded as having major protocol violations were higher than expected and halved the total number of infants in the PP population. This may have been due to the strict exclusion criteria set for the PP population. Hence, the study was underpowered to demonstrate equivalence in weight gain in the PP population, which would have required at least sixty-nine children per treatment group (based on *α* = 0·05, sd = 6 g/d, equivalence margin of 0·5 sd and a power of 0·80), instead of the forty-five and fifty-seven infants present in the synbiotic group and control group, respectively. Lastly, the stool and blood samples were only collected at four of the seventeen sites, since this would result in a sufficient amount of samples required for the detection of clinically relevant differences in serum values as well as microbiota composition and faecal metabolic profile. This pre-selection could have induced some bias since this selected subgroup might not have been a proper reflection of the total population with respect to these outcome parameters.

In conclusion, this study demonstrates, based on both the equivalence of growth in the ITT population as well as the close to zero weight-for-age *z*-scores in both the ITT and PP populations compared with the WHO growth standard, an adequate growth of infants consuming an extensively hydrolysed formula containing scGOS/lcFOS (9:1) and *B. breve* M-16V. No differences were observed in the number of adverse events compared with a control formula. Moreover, the previously observed effects on faecal microbiota composition and associated metabolic profile were to a large extent confirmed in this study. This demonstrates that there are no safety concerns for the addition of both scGOS/lcFOS (9:1) and *B. breve* M-16V to extensively hydrolysed milk formulas intended for young, healthy infants.
